# Supplementary material for: Cloud BioLinux: pre-configured and on-demand bioinformatics computing for the genomics community
Source: BMC Bioinformatics. 2012 Mar 19;13:42. doi: 10.1186/1471-2105-13-42 (PMC3372431; doi:10.1186/1471-2105-13-42)
Supplement: Additional file 1 — Supplementary 1 Cloud BioLinux software documentation in the form of a mini, self-contained website. Users need to download and uncompress the .zip file, and open through a web browser the "index.html" file available on the main directory. (ZIP 1823 kb). [file 1471-2105-13-42-S1.ZIP › Cloud-BioLinux-Package-Documentation/docs/bwa.html]

Bio-Linux Software Documentation Pages

Back to search form

## bwa

|  |  |
| --- | --- |
| Name | bwa |
| Description | Burrows-Wheeler Aligner (bwa) is a program that aligns relatively short nucleotide sequences against a long reference sequence such as the human genome. It implements two algorithms, bwa-short, which works for query sequences shorter than 200 bp and BWA-SP which works for longer sequences, up to around 100 kbp. Both algorithms do gapped alignment. They are usually more accurate and faster on queries with low error rates.  The BWA package is prepared by Debian-Med and can be installed in Bio-Linux by typing: `sudo apt-get install bwa` For further information about BWA refer to the remote documentation |
| Homepage | http://bio-bwa.sourceforge.net/ |
| Remote Documentation | http://bio-bwa.sourceforge.net/bwa.shtml |
